# Swine-Derived Probiotic *Lactobacillus plantarum* Modulates Porcine Intestinal Endogenous Host Defense Peptide Synthesis Through TLR2/MAPK/AP-1 Signaling Pathway

**DOI:** 10.3389/fimmu.2019.02691

**Published:** 2019-11-19

**Authors:** Jing Wang, Wei Zhang, Sixin Wang, Hui Liu, Dongyan Zhang, Yamin Wang, Haifeng Ji

**Affiliations:** Institute of Animal Husbandry and Veterinary Medicine, Beijing Academy of Agriculture and Forestry Sciences, Beijing, China

**Keywords:** *Lactobacillus plantarum* ZLP001, host defense peptide, toll-like receptor 2, mitogen-activated protein kinase, activator protein 1, weaning piglet

## Abstract

Host defense peptides (HDPs) have antimicrobial and immunoregulatory activities and are involved in epithelial innate immune defense. Dietary modulation of endogenous HDP synthesis is an effective way to boost the host innate immune system. This study aimed to investigate the role of the probiotic *Lactobacillus plantarum* strain ZLP001 in porcine HDP induction and the underlying mechanism. To this end, we evaluated the stimulatory effect of *L. plantarum* ZLP001 on HDP expression in piglet intestinal tissue *in vivo* and porcine IPEC-J2 cells and 3D4/31 cells *in vitro*, and we examined the underlying intracellular signaling pathway in IPEC-J2 cells. *L. plantarum* ZLP001 treatment increased the mRNA expression of jejunal and ileal HDPs in weaned piglets. In IPEC-J2 and 3D4/31 cells, *L. plantarum* ZLP001 stimulated HDP expression, but different HDP induction patterns were observed, with the various HDPs exhibiting different relative mRNA levels in each cell line. *L. plantarum* ZLP001 induced porcine HDP expression through toll-like receptor (TLR)2 recognition as evidenced by the fact that HDP expression was suppressed in TLR2-knockdown IPEC-J2 cells. Furthermore, we found that *L. plantarum* ZLP001 activated the extracellular signal-regulated kinase (ERK)1/2 and c-jun N-terminal kinase (JNK) signaling pathways, as indicated by enhanced phosphorylation of both ERK1/2 and JNK and the fact that HDP expression was suppressed upon inhibition of ERK1/2 and JNK. Furthermore, *L. plantarum* ZLP001 activated c-fos and c-jun transcription factor phosphorylation and activity. We conclude that *L. plantarum* ZLP001 induces porcine HDP expression *in vivo* and *in vitro*, and the induction seems to be regulated *via* TLR2 as well as the ERK1/2/JNK and c-jun/c-fos signaling pathways. Modulation of endogenous HDPs mediated by *L. plantarum* ZLP001 might be a promising approach to improving intestinal health and enhancing diarrhea resistance in weaning piglets.

## Introduction

Weaning stress in piglets can suppress immune responses, rendering the piglets vulnerable to infectious diseases, including diarrhea (1). Since mid-last century, antibiotics have been widely used at subtherapeutic levels in piglet production for disease control and growth promotion. The overuse of antibiotics has led to the development of antimicrobial-resistant bacterial strains, which threaten animal as well as human health. Because of this public safety concern, antibiotic additives will be phased out in China, as in many other countries. Therefore, novel methods to inhibit pathogen overgrowth in piglets and improve their host defense responses are urgently needed (2, 3).

Host defense peptides (HDPs), also known as antimicrobial peptides, are produced in various epithelial cells and immune cells lining the gut and play essential roles in mammalian innate immunity. HDPs have two main families (i.e., defensins and cathelicidins) in vertebrate animals. They have been widely studied for their broad-spectrum antimicrobial effects against bacteria, viruses, fungi, and protozoa (4). In addition to the well-described antimicrobial properties, HDPs display a wide range of immunomodulatory activities, including modulation of pro-, and anti-inflammatory responses (4, 5). Stimulating the innate immune system and elevating endogenous HPD synthesis through dietary supplementation has been suggested as a possible safe method to enhance bacterial balance and improve intestinal health (6, 7). In normal physiological conditions, HDPs are activated in response to the oscillating energy status of cells and tissues and help maintain and enhance defensive barriers without causing damage or inflammation (8). Certain nutritional components, such as VD_3_, butyrate, and zinc, induce HDP gene expression in animals, and humans without causing inflammatory damage (9–11). Likewise, certain probiotic strains can also induce the release of HDP from epithelial cells (12, 13), which may contribute to explain the beneficial effects of probiotics in infection preventing and gut barrier function stabilizing. Some human research suggests that toll-like receptors (TLRs) might participate in the regulating of probiotic associate HDPs induction effect, and this induction might also be mediated by proinflammatory pathways (14). However, the extent of modulation could be varied in different models and among different strains (15).

In our previous study, we demonstrated that *Lactobacillus plantarum* ZLP001, which we isolated from healthy piglet ileal mucosa (16), exert beneficial effects on growth performance and antioxidant status in weaning piglets (17), also can upregulate the expression of HDPs in IPEC-J2 cells but did not trigger an inflammatory response (18). However, little is known about the stimulatory effects of *L. plantarum* ZLP001 on macrophages and *in vivo*. Additionally, the mechanisms and possible cellular pathways of probiotic-mediated HDP induction have been investigated only in humans, never in pigs. In this study, we examined whether *L. plantarum* ZLP001 treatment stimulates HDP gene expression in weaning piglets. Additionally, different porcine cell lines, including 3D4/31 lung alveolar macrophages, were used to evaluate the potential regulatory effects of *L. plantarum* ZLP001 on HDP expression. Furthermore, a potential signaling pathway responsible for *L. plantarum* ZLP001 modulation of HDP expression was investigated.

## Materials and Methods

### Animals, Diets, and Experimental Design

Twenty piglets (Large White × Landrace) weaned at 28 days of age (body weight, 8.11 ± 0.35 kg) were used in this study. The experiment was conducted at the experimental animal farm of the Institute of Animal Husbandry and Veterinary Medicine of the Beijing Academy of Agriculture and Forestry Sciences (IAHVM-BAAFS) according to the guidelines established by the Animal Care and Use Committee of IAHVM-BAAFS. The protocol approval number was XM1706. The animals were given humane care throughout the trial. The piglets were raised in a room that was decontaminated prior to the study and maintained at 25–28°C. All piglets had free access to feed and water throughout the 28-days experimental period. They received a complete feed specially formulated according to the National Research Council (19) and the Feeding Standard of Swine (20). The ingredients and chemical composition of the diet are presented in Table 1. The experiment was designed as a contrast experiment including two dietary treatments, and piglets were randomly assigned to one of the treatments (10 piglets per treatment). In the control group, the basal diet was supplemented with a placebo (2 g/kg diet), and in the treatment group, it was supplemented with freeze-dried *L. plantarum* ZLP001 (10^9^ CFU/g, 2 g/kg diet). The concentration of supplemented *L. plantarum* ZLP001 in the diet was determined based on the findings of our previous studies (16, 17) and post-extension practice results on the farm. On day 28, the piglets were euthanized. Segments of about 5-cm middle tissues from the duodenum, jejunum, and ileum were collected, rinsed with 0.9% (w/v) saline solution, immediately frozen in liquid nitrogen, and then stored at −80°C until use.

**Table 1:** Ingredients and chemical composition of the basal diet.

**Ingredient**	**Content (g/kg)**
Expanded corn	532
Soybean meal	220
Wheat bran	50
Fish meal	20
Peeling soybean meal	70
Whey powder	50
Soybean oil	10
Complex enzyme	0.5
Choline	0.1
Limestone	0.2
Premix^a^	40
Chemical compositions	
Digestible energy^b^ (MJ kg^−1^)	13.9
Crude protein^c^	200.5
Lysine^c^	12.3
Methionine^c^	3.2
Calcium^c^	8.0
Total phosphorus^c^	3.2

a *Provided per kg of complete diet: vitamin A, 10,500 IU; vitamin D_3_, 3,500 IU; vitamin E, 35 mg; niacin, 30 mg; pantothenic acid, 12.0 mg; riboflavin, 6.5 mg; menadione, 2.0 mg; vitamin B_12_, 0.045 mg; Fe, 145 mg; Zn, 130 mg; Cu, 120 mg; Mn, 35 mg; I, 0.5 mg; Se, 0.2 mg*.

b *Calculated nutrient levels*.

c *Measured nutrient levels*.

### Probiotic Strain and Culture Conditions

*L. plantarum* ZLP001, originally isolated in our laboratory from ileal mucosa of healthy piglets 4 weeks after weaning, has been identified by the China Center of Industrial Culture Collection (Beijing, China) and is preserved in the China General Microbiological Culture Collection Center (CGMCC No. 7370). After thawing and overnight culture, *L. plantarum* ZLP001 was inoculated 1:100 in improved de Man, Rogosa, and Sharpe (MRS) liquid medium (10 g peptone, 5 g yeast powder, 20 g glucose, 10 g beef extract, 5 g sodium acetate, 2 g ammonium citrate dibasic, 2 g dipotassium phosphate, 0.58 g magnesium sulfate, 0.19 g manganese sulfate, 1 ml of Tween-80, and water to 1,000 ml; pH 6.5) and incubated at 37°C under anaerobic conditions for 18 h. For probiotic preparation, bacterial cells were collected by centrifugation at 10,000 × *g* for 10 min, the supernatant was discarded, and the bacterial sludge was mixed with protective medium (100 ml contained 12 g skimmed milk, 10 g dextrin, 4 g lactose, 1.5 g sodium glutamate, and 0.5 g l-cysteine). The mixture was vortexed for 30 min, added to a freeze-drying plate, incubated at −70°C for 2 h, and dried in a freeze-dryer at 1 Pa and −80°C for 24 h. The viable cell count of the freeze-dried powder was 10^9^ CFU/g. The negative placebo sample consisted of protective medium without *L. plantarum* ZLP001 subjected to the same procedure.

### Cell Lines and Culture Conditions

The porcine intestinal epithelial cell line IPEC-J2, which was originally derived from jejunal tissues of neonatal piglets (21), was kindly provided by Dr. Glenn Zhang at Oklahoma State University (Stillwater, OK, USA). IPEC-J2 cells were cultured in Dulbecco's modified Eagle medium/Nutrient Mixture F-12 (DMEM/F12; a 1:1 mixture of DMEM and Ham's F-12; Invitrogen, Carlsbad, CA, USA) supplemented with 10% fetal bovine serum (FBS; Invitrogen), streptomycin (100 μg/ml), penicillin (100 U/ml), and 1% insulin, transferrin, selenium (ScienCell, San Diego, CA, USA) at 37°C under a 5% CO_2_ and 95% air atmosphere with 90% humidity.

The porcine lung alveolar macrophage cell line 3D4/31 (ATCC CRL-2844) was also kindly provided by Dr. Zhang. The 3D4/31 cells were cultured in RPMI 1640 medium (Invitrogen, Carlsbad, CA, USA) supplemented with 10% FBS, 100 μg/ml streptomycin, 100 U/ml penicillin, and 1 mM sodium pyruvate at 37°C under a 5% CO_2_ and 95% air atmosphere with 90% humidity.

### Treatment of IPEC-J2 and 3D4/31 Cells With *L. plantarum* ZLP001

To prevent any influence of the FBS and antibiotics on the immune response, bacteria were collected by centrifugation and diluted in fresh DMEM/F12 without FBS and streptomycin/penicillin. The cells were seeded in six-well tissue-culture plates (Costar, Corning Inc., Corning, NY, USA) at 2.5 × 10^5^ cells/well for IPEC-J2 cells and 1 × 10^6^ cells/well for 3D4/31 cells. When the cells reached 70–80% confluence, they were exposed to *L. plantarum* ZLP001 (10^8^ CFU/ml) in duplicate for 6 h. We used live *L. plantarum* ZLP001 in the present study, which exhibits the highest induction effect on HDP expression compared with other bacteria processing (heat-killed *L. plantarum* ZLP001, adhered *L. plantarum* ZLP001, undirect contact *L. plantarum* ZLP001, and bacterial free culture supernatant of *L. plantarum* ZLP001) (data not shown). The optimal bacteria concentration and treatment time were selected from our previous study based on a concentration-dependent (10^5^, 10^6^, 10^7^, 10^8^, and 10^9^ CFU/ml *L. plantarum* ZLP001) and a time-dependent (3, 6, 9, and 12 h) experiment (18). Non-treated cells served as a control. After incubation, the cells were washed with PBS three times and collected for further assay. The culture media were also collected. All experiments were carried out in triplicate.

The involvement of different mitogen-activated protein kinase (MAPK) signaling pathways in *L. plantarum* ZLP001-induced expression of HDPs at both the transcriptional and translational levels was evaluated by using specific pharmacological inhibitors. The specific extracellular signal-regulated kinase (ERK)1/2 and c-jun N-terminal kinase (JNK) inhibitors U0126 and SB600125 were purchased from Cell Signaling Technology (Danvers, MA, USA) and were dissolved in dimethyl sulfoxide. IPEC-J2 cells were pretreated with the specific inhibitors for 1 h prior to stimulation with *L. plantarum* ZLP001. After the indicated incubation periods, cell samples were collected to examine mRNA expression by RT-qPCR and cell culture media were collected to analyze protein secretion by ELISA. Appropriate inhibitor concentration and incubation time were evaluated by Cell Counting Kit-8 (Dojindo Molecular Technologies, Kumamoto, Japan). All experiments were carried out in triplicate.

### Quantitative Reverse Transcription (RT-q)PCR

HDP mRNA levels were determined by RT-qPCR. Cells and tissues were lysed directly in RNAzol (Molecular Research Center, Cincinnati, OH, USA), and total RNA was extracted according to the manufacturer's instructions. The RNA concentration was measured using a NanoDrop spectrophotometer (Thermo Fisher Scientific, Waltham, MA, USA), and the quality was ascertained by the A260:A280 and A260:A230 ratios. One microgram of RNA was reverse-transcribed to cDNA using an iScript™ cDNA Synthesis Kit (Bio-Rad, Hercules, CA, USA) according to the manufacturer's instructions. qPCR was conducted using iTaq™ Universal SYBR® Green Supermix (Bio-Rad) in a QuantStudio 3 Real-Time PCR System (Thermo Fisher Scientific). The thermal cycles were as follows: 95°C for 10 min followed by 40 cycles of 95°C for 30 s, 60°C for 30 s, and 72°C for 20 s. Porcine-specific primers were designed, and the sequences are listed in Supplementary Table S1. Target gene expression levels were normalized to that of glyceraldehyde-3-phosphate dehydrogenase (GAPDH), and relative gene expression was calculated using the ΔΔCt method.

### Western Blotting

Proteins were extracted from IPEC-J2 cells using lysis buffer consisting of 150 mM NaCl, 1% Triton X-100, 0.5% sodium deoxycholate, 0.1% sodium dodecyl sulfate, 50 mM Tris-HCl at pH 7.4, and a protease inhibitor cocktail (Applygene, Beijing, China). Cells were mixed with 100 μl of ice-cold lysis buffer and incubated on ice for 30 min. The mixtures were centrifuged at 12,000 × *g* at 4°C for 5 min, and protein concentrations in the supernatants were determined using a BCA Protein Assay Kit (Pierce, Madison, WI, USA). Equal amounts of protein (30 μg/lane) were resolved by 10% sodium dodecyl sulfate polyacrylamide gel electrophoresis and electrophoretically transferred to polyvinylidene difluoride membranes (EMD Millipore, Bedford, MA, USA) at 90 V at 4°C for 60–100 min. After being blocked with 5% skim milk, the immunoblots were incubated with primary antibodies at 4°C overnight, then with the indicated horseradish peroxidase-conjugated secondary antibodies, and finally reacted with Western Blot Luminance Reagent (Santa Cruz Biotechnology, Dallas, TX, USA). The antibodies and dilutions used are shown in Supplementary Table S2. Immunoreactive proteins were imaged with a ChemiDoc XRS system (Bio-Rad, Hercules, CA, USA). Band densities were quantified using ImageJ (National Institutes of Health, Bethesda, MD, USA) and normalized to that of the loading control.

### Enzyme-Linked Immunosorbent Assay (ELISA)

Five hundred microliters of cell culture media were centrifuged at 4,000 × *g* at room temperature for 10 min and then passed through 0.25-μm filters (Corning Inc., Corning, NY, USA). The concentration of porcine β-defensin 2 (pBD2) was determined using a commercial porcine-specific ELISA kit (CloudClone Corp. USCN Life Science, Inc., Wuhan, China) according to the manufacturer's instructions. We also tried to detect the concentration of pBD2 in the intestinal content and mucus, but we failed to get the valid data.

### TLR2 Knockdown in IPEC-J2 Cells

TLR2 was knocked down in IPEC-J2 cells according to the method described by Murofushi et al. (22). IPEC-J2 cells were seeded in six-well tissue culture plates at 2.5 × 10^5^ cells/well. When the cells reached 70–80% confluence, they were transfected with 300 pmol of TLR short interfering RNAs (siRNAs) using Lipofectamine RNAiMAX (Invitrogen, Carlsbad, CA, USA). The primers used for TLR2 gene knockdown were as follows: CAGAUGCCUCCUUUCUACCCAUGUU (sense) and AACAUGGGUAGAAAGGAGGCAUCUG (antisense). Inhibition of TLR2 expression in knockdown IPEC-J2 cells was detected by qPCR with TLR2-specific primers (sense: TTCAGGCCAAGGATTTCCAG; antisense: TCACTGTGCTGGTTCATTG).

### Transcription Factor Activity Assay

DNA binding by c-jun and c-fos was detected using the TransAM transcription factor ELISA system (Active Motif, Carlsbad, CA, USA) according to the manufacturer's instructions. After stimulation with *L. plantarum* ZLP001, nuclear proteins were isolated from IPEC-J2 cells using a nuclear protein extraction kit (Active Motif, Carlsbad, CA, USA) according to the manufacturer's instructions. Ten micrograms of IPEC-J2 cell nuclear extract was incubated in oligonucleotide-coated wells at room temperature for 20 min. The plates were washed with PBS three times, and 100 μl of the corresponding transcription factor antibody (1:1,000) was added to each well and incubated for 1 h. After three washes with PBS, 100 μl of horseradish peroxidase-conjugated antibody (1:1,000) was added for 1 h. The plates were then incubated with developing solution for an additional 10 min, and the absorbance at 450 nm was measured using a spectrophotometer.

### Statistical Analysis

Statistical analyses were performed using Prism version 6 (GraphPad Software, Inc., San Diego, CA, USA). Statistical significance between different treatments was determined using unpaired Student's two-tailed *t*-test. The results are expressed as means ± standard error of the mean (SEM). The minimal level of confidence at which experimental results were considered significant was set at *P* < 0.05.

## Results

### *L. plantarum* ZLP001 Stimulates Intestinal HDP Expression *in vivo*

**Figure 1:**
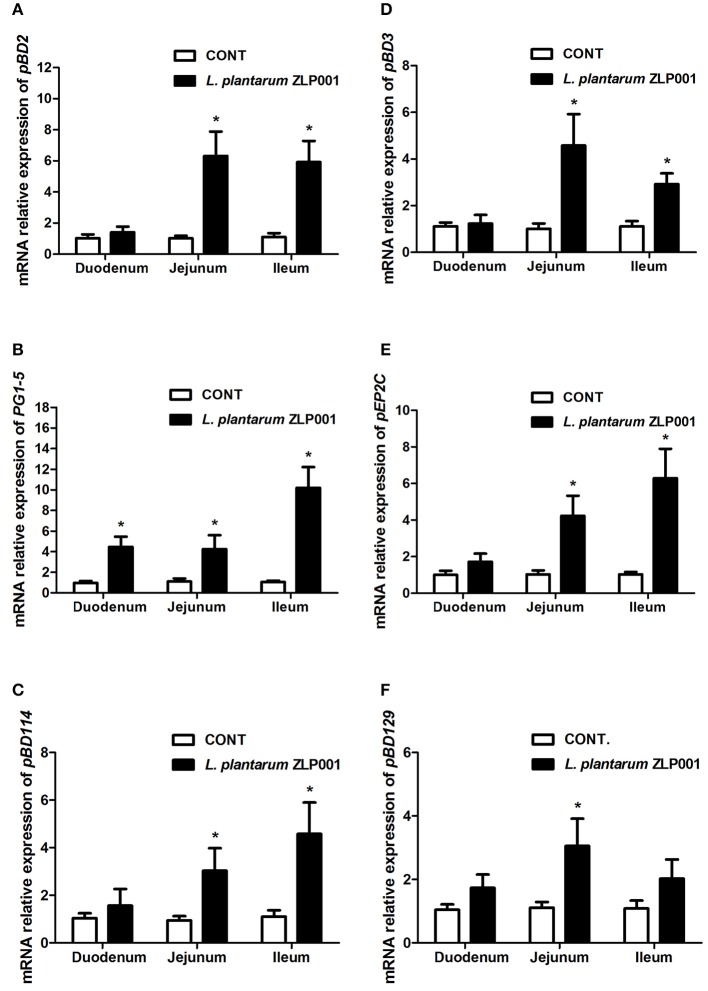
Relative mRNA expression of porcine host defense peptides (HDPs) in the duodena, jejuna, and ilea of piglets supplemented with *L. plantarum* ZLP001 for 4 weeks as determined by RT-qPCR. Average Ct values of each gene in duodenum, jejunum, and ileum were **(A)** *pBD2* 31.0/30.6, 30.9/28.6, 30.4/27.9; **(B)** *PG1-5* 30.8/28.8, 29.6/28.0, 31.2/27.5; **(C)** *pBD114* 31.1/31.0, 30.8/29.8, 31.9/30.3; **(D)** *pBD3* 30.5/30.4, 29.8/27.8, 29.8/29.4; **(E)** *pEP2C* 30.7/29.9, 31.5/29.4, 30.9/28.7; and **(F)** *pBD129* 30.2/30.3, 29.9/28.4, 31.0/29.9, respectively. mRNA expression was standardized to that of GAPDH. Relative fold changes vs. levels in non-stimulated controls were calculated by the ΔΔCt method. Data are the mean ± SEM of three independent experiments. *P < 0.05 vs. non-treated control group. White and black bars represent control and *L. plantarum* ZLP001 treatment, respectively.

*L. plantarum* ZLP001 was administered to weaned piglets at 2 g/kg diet for 28 days to determine whether it affects HDP expression in intestinal tissues. mRNA expression of six porcine HDPs, including *pBD2, pBD3, PG1–5* (cysteine-rich protegrins 1–5), *pEP2C* (epididymis protein 2 splicing variant C), *pBD114*, and *pBD129*, was evaluated by RT-qPCR (Figure 1). Except for *PG1-5*, HDP mRNA expression levels in the duodenum did not differ (*P* > 0.05) between the *L. plantarum* ZLP001 treatment and control groups. For most HDPs, intragroup differences in mRNA expression levels in the duodenum were greater than intergroup differences. In the jejunum, dietary supplementation with *L. plantarum* ZLP001 resulted in significantly enhanced expression of the six HDPs (*P* < 0.05 vs. control group). In ileal tissues, *L. plantarum* ZLP001 supplementation resulted in significantly enhanced mRNA expression of all HDPs evaluated except *pBD129*. The evaluated genes responded differentially to *L. plantarum* ZLP001. in different intestinal tissue types. In the jejunum, *pBD2* showed the strongest response, with an ~6-fold increase, whereas the other HDPs showed intermediate 3–4-fold induction. In the ileum, *PG1-5* exhibited the strongest response, with an ~10-fold increase, and *pBD2, pBD114*, and *pEP2C* exhibited higher fold increases than did *pBD3*, which were only slightly induced (<3-fold).

### *L. plantarum* ZLP001 Induces HDP Expression in IPEC-J2 and 3D4/31 Cells *in vitro*

**Figure 2:**
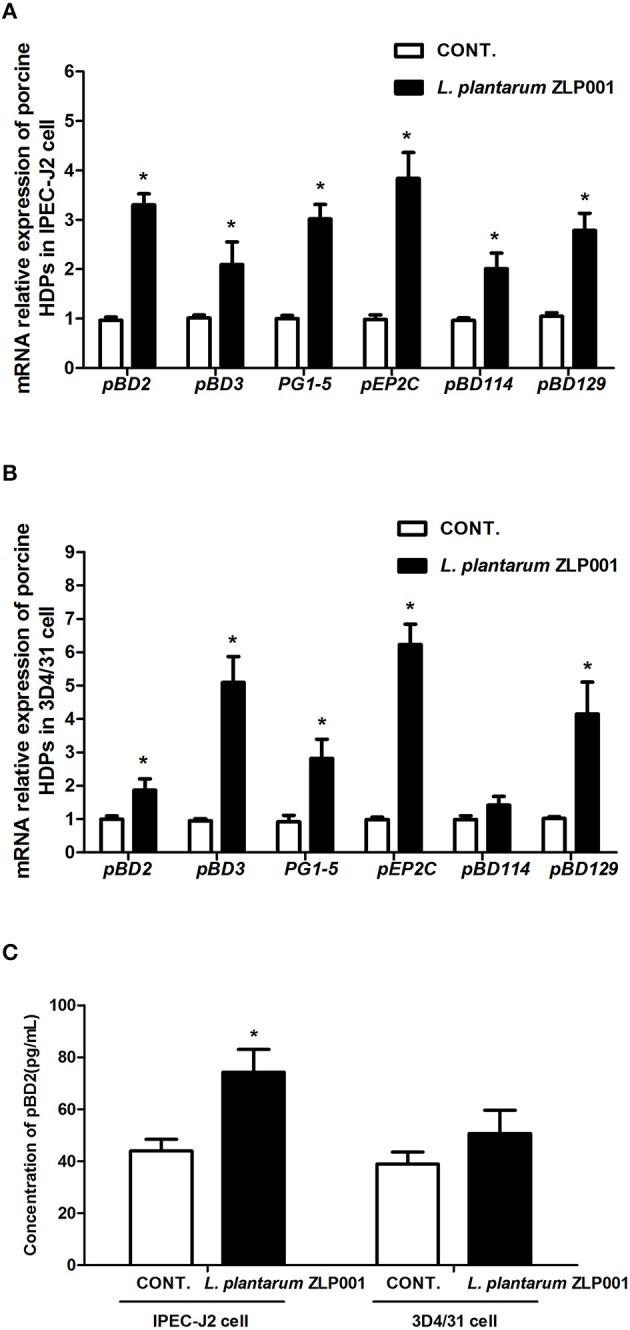
Relative mRNA expression and protein secretion of host defense peptides (HDPs) in different porcine cell lines after *L. plantarum* ZLP001 treatment. mRNA expression in **(A)** intestinal IPEC-J2 epithelial cells and **(B)** 3D4/31 lung alveolar macrophages as determined by RT-qPCR. Average Ct values of each gene in IPEC-J2 cell and 3D4/31 cell were **(A)** *pBD2* 31.7/29.6, *pBD3* 29.1/28.0, *PG1-5* 31.9/30.1, *pEP2C* 30.5/28.6, *pBD114* 32.0/31.0, *pBD129* 31.3/29.8; and **(B)** *pBD2* 30.3/29.5, *pBD3* 30.6/27.9, *PG1-5* 31.3/29.5, *pEP2C* 29.7/26.9, *pBD114* 31.2/30.7, *pBD129* 30.7/28.7. mRNA expression was standardized to that of GAPDH. Relative fold changes vs. levels in non-stimulated controls were calculated by the ΔΔCt method. **(C)** pBD2 secretion in both cell lines after *L. plantarum* ZLP001 treatment as determined by ELISA. Data are the mean ± SEM of three independent experiments. *P < 0.05 vs. non-treated control group. White and black bars represent control and *L. plantarum* ZLP001 treatment, respectively.

First, we analyzed the mRNA expression of the above six porcine HDPs in IPEC-J2 intestinal epithelial cells exposed to *L. plantarum* ZLP001 by RT-qPCR. After 6-h exposure to *L. plantarum* ZLP001, mRNA expression of all six HDPs was significantly increased (Figure 2A). However, the magnitude of induction obviously differed among the six HDPs; *pEP2C* showed an ~4-fold increase; *pBD2, PG1-5*, and *pBD129* exhibited ~3-fold induction; and *pBD3* and *pBD114* showed lower induction.

Next, we evaluated the effect of *L. plantarum* ZLP001 on porcine 3D4/31 lung alveolar macrophages (Figure 2B). mRNA levels of *pBD2, pBD3, PG1-5, pEP2C*, and *pBD129* were significantly increased after incubation with *L. plantarum* ZLP001. The induction patterns of several genes in IPEC-J2 and 3D4/31 cells were in sharp contrast. *pBD3* was substantially more strongly induced in 3D4/31 cells, with a nearly five-fold increase following *L. plantarum* ZLP001 treatment, whereas *pBD2* exhibited a <2-fold increase. *PG1-5* induction was similar in both cell lines. *pEP2C* expression exhibited a six-fold increase in 3D4/31 cells following *L. plantarum* ZLP001 treatment. *pBD114* was not significantly induced in 3D4/31 cells.

To evaluate the effect of *L. plantarum* ZLP001 on HDP secretion, we examined the concentration of pBD2 in cell culture media after *L. plantarum* ZLP001 treatment (commercial ELISA kits for other HDPs were unavailable). As shown in Figure 2C, *L. plantarum* ZLP001 significantly induced pBD2 production in IPEC-J2 but not 3D4/31 cells.

### TLR2 Is Required for *L. plantarum* ZLP001-Induced HDP Upregulation in IPEC-J2 Cells

**Figure 3:**
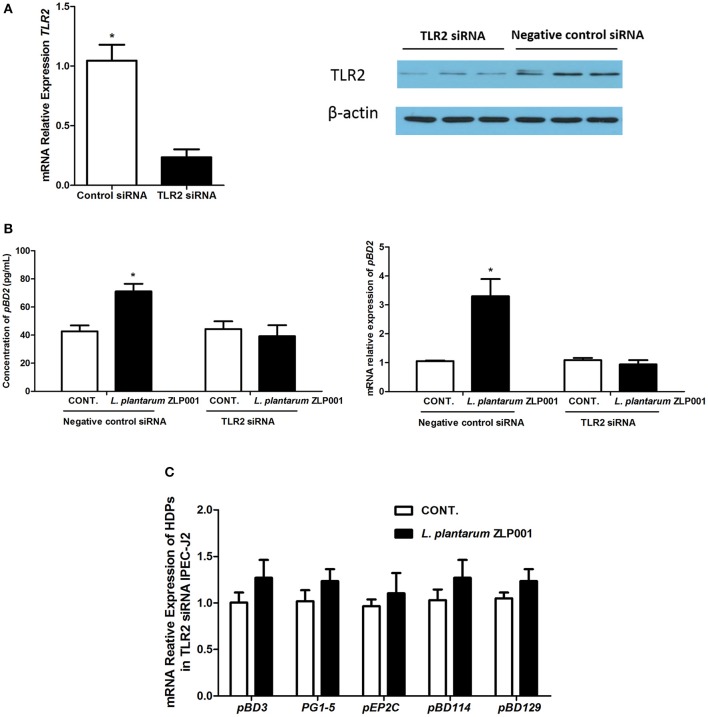
Toll-like receptor (TLR)2 is required for *L. plantarum* ZLP001-induced porcine host defense peptide (HDP) upregulation in IPEC-J2 cells. **(A)** *TLR2* gene and protein expression in TLR2 siRNA-transfected IPEC-J2 cells was determined using RT-qPCR and Western blot analyses, respectively. Average Ct values of *TLR2* gene were 30.3/32.7. **(B)** *L. plantarum* ZLP001 stimulates porcine pBD2 expression and secretion through TLR2 in IPEC-J2 cells. pBD2 expression and concentration were measured by RT-qPCR and ELISA, respectively. Average Ct values of *pBD2* gene were 31.0/29.5 and 32.4/32.7 for negative control siRNA and TLR2 siRNA, respectively. Data are the mean ± SEM of three independent experiments. *P < 0.05 vs. non-treated control group. **(C)** TLR2 silencing suppresses porcine HDP expression induced by *L. plantarum* ZLP001 in IPEC-J2 cells. Average Ct values of each gene were *pBD3* 30.3/29.7, *PG1-5* 30.4/30.6, *pEP2C* 30.0/30.4, *pBD114* 31.1/30.3, *pBD129* 30.2/29.8, respectively.

TLR2 is involved in the cellular response to probiotic bacteria (23). To examine whether TLR2 is involved in the upregulation of HDP expression by *L. plantarum* ZLP001 treatment, IPEC-J2 cells were transfected with siRNA to specifically knock down TLR2. Effective knockdown of TLR2 was confirmed by RT-qPCR and Western blot analysis (Figure 3A). *pBD2* mRNA expression and pBD2 protein secretion were significantly decreased in TLR2-knockdown IPEC-J2 cells when compared with negative siRNA control cells after *L. plantarum* ZLP001 treatment, as indicated by RT-qPCR and Western blot analysis (Figure 3B). mRNA levels of the other HDPs were also suppressed in TLR2-knockdown cells treated with *L. plantarum* ZLP001 (Figure 3C). These results indicated that TLR2 is required for *L. plantarum* ZLP001-induced HDP upregulation in IPEC-J2 cells.

### *L. plantarum* ZLP001-Induced HDP Expression Is Regulated by MAPK Signaling in IPEC-J2 Cells

**Figure 4:**
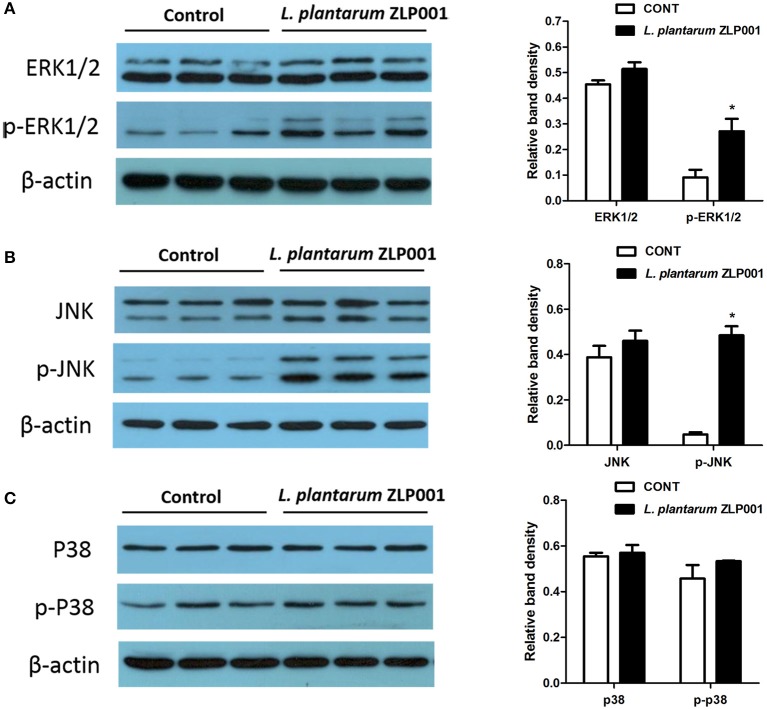
Role of mitogen-activated protein kinase (MAPK) signaling pathways in *L. plantarum* ZLP001-induced porcine host defense peptide (HDP) expression and secretion. IPEC-J2 cells were incubated with *L. plantarum* ZLP001 at 10^8^ CFU/ml for 6 h, and protein expression and phosphorylation of extracellular signal-regulated kinase (ERK)1/2 **(A)**, ERK **(B)**, and p38 **(C)** in whole-cell lysates were assessed by Western blot analysis.

Activation of MAPK has been associated with HDP expression (12). Western blot analysis revealed increased ERK1/2 and JNK phosphorylation but not p38 phosphorylation after *L. plantarum* ZLP001 treatment (Figure 4), suggesting that *L. plantarum* ZLP001 activated both the ERK1/2 and JNK signaling pathways.

**Figure 5:**
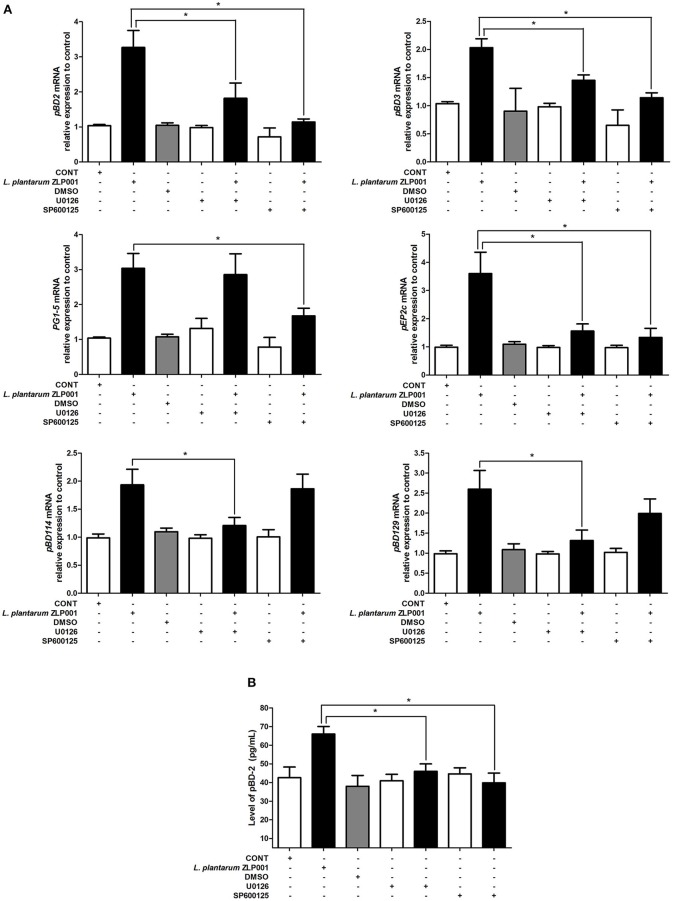
Blocking the key proteins of mitogen-activated protein kinase (MAPK) signaling pathway affects porcine host defense peptide (HDP) expression and production. **(A)** Inhibition of extracellular signal-regulated kinase (ERK)1/2 and c-jun N-terminal kinase (JNK) blocks porcine HDP mRNA expression. IPEC-J2 cells were preincubated with the specific ERK1/2 inhibitor U0126 (10 μM) and the specific JNK inhibitor SP600125 (10 μM) 1 h before incubation with 10^8^ CFU/ml *L. plantarum* ZLP001 for 6 h. Average Ct values of each gene were *pBD2* 30.9/29.3/31.6/31.1/30.0/31.5/30.5, *PG1-5* 30.1/28.5/29.9/30.2/28.2/31.0/29.3, *pBD114* 31.6/30.5/31.3/32.0/31.2/31.3/30.5, *pBD3* 30.3/29.1/31.6/30.0/29.4/31.1/29.9, *pEP2C* 30.1/28.7/29.7/30.2/29.6/30.7/30.3, and *pBD129* 30.7/29.0/30.1/30.6/30.0/30.4/29.4, respectively. **(B)** Inhibition of ERK1/2 and JNK blocks porcine pBD2 production. The concentration of pBD2 in the supernatant of *L. plantarum* ZLP001-treated IPEC-J2 cells was analyzed by ELISA. Gray level, mRNA expression data, and ELISA data are presented as the mean ± SEM of three independent experiments. *P < 0.05 vs. non-treated control group.

We also examined whether these two pathways are required for *L. plantarum* ZLP001-induced porcine HPD expression using specific inhibitors (Figure 5A). Neither the ERK1/2 inhibitor U0126 nor the JNK inhibitor SP600125 significantly induced HDP expression in IPEC-J2 cells. U0126 and SP600125 treatments partially suppressed the increases in *pBD2, pBD3*, and *pEP2C* mRNA expression induced by *L. plantarum* ZLP001. U0126 failed to inhibit *PG1-5* induction and SP600125 failed to inhibit *pBD114* and *pBD129* induction by *L. plantarum* ZLP001 at the mRNA level. These results suggested that different HDPs are regulated by different signaling pathways.

When we measured pBD2 secretion by IPEC-J2 cells upon U0126 and SP600125 treatment, we found that pBD2 concentrations in culture media were significantly decreased after inhibitor treatment compared with those in cells treated with *L. plantarum* ZLP001 alone (Figure 5B). These findings suggested that pBD2 production induced by *L. plantarum* ZLP001 was blocked by the ERK1/2 and JNK inhibitors.

### Activator Protein (AP)-1 Regulates *L. plantarum* ZLP001-Induced HDP Upregulation in IPEC-J2 Cells

**Figure 6:**
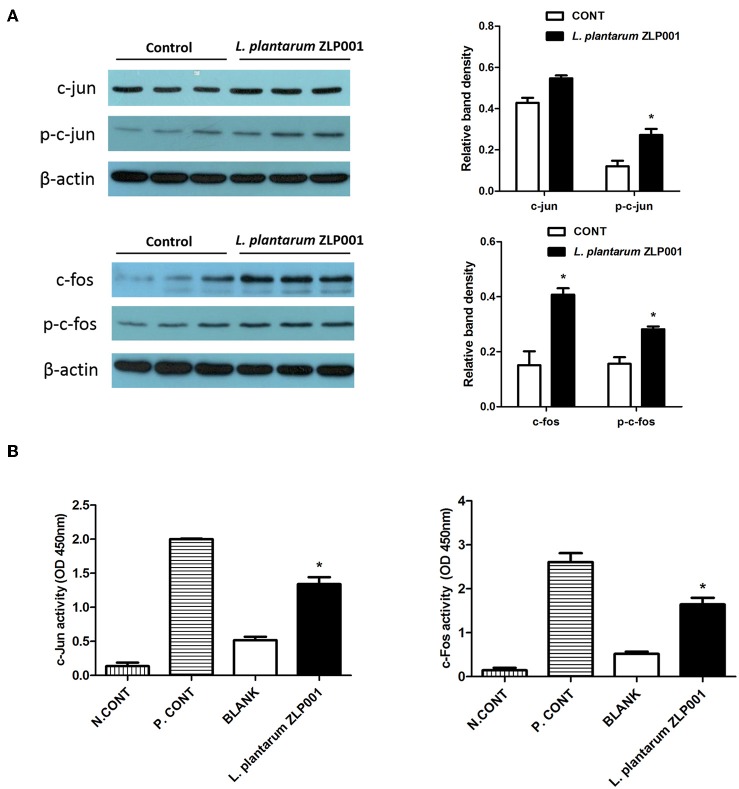
Role of transcription factor mitogen-activated protein kinase (MAPK)/activator protein (AP)-1 in *L. plantarum* ZLP001-induced host defense peptide (HDP) expression. **(A)** *L. plantarum* ZLP001 induces c-fos and c-jun protein expression. IPEC-J2 cells were incubated with 10^8^ CFU/ml *L. plantarum* ZLP001 for 6 h, and protein expression and phosphorylation of c-jun and c-fos were assessed by Western blot analysis in whole-cell lysates. **(B)** *L. plantarum* ZLP001 increased AP-1 subunit c-jun and c-fos activities. c-jun and c-fos activities in nuclear extracts were assessed by TransAM assay. A nuclear extract provided from the supplier served as a positive control, and a negative control was incubated without nuclear extract. Gray level and activity data are presented as the mean ± SEM of three independent experiments. *P < 0.05 vs. non-treated control group.

The AP-1 signaling pathway plays an important role in the innate immune response. AP-1 is composed of hetero- and homodimers of c-jun and c-fos family members (24). Therefore, we evaluated whether *L. plantarum* ZLP001-induced HDP upregulation is regulated by c-fos and c-jun, which are the main downstream effectors activated by ERK1/2 and JNK signaling. Treatment of IPEC-J2 cells with *L. plantarum* ZLP001 for 6 h increased protein expression of c-fos and c-jun in IPEC-J2 cells (Figure 6A). In addition, c-fos and c-jun activities were induced by *L. plantarum* ZLP001 when compared to the levels in non-treated IPEC-J2 cells (*P* < 0.05; Figure 6B). These results suggested a role for c-fos and c-jun in regulating the stimulatory effects of *L. plantarum* ZLP001 on HDP expression in IPEC-J2 cells.

## Discussion

The mucosal immune system plays an important role in maintaining gastrointestinal function in pigs (25). The intestinal epithelium of piglets is immature and is weakened under weaning stress. The establishment of a strong gastrointestinal immune system after weaning is crucial to enhancing tolerance to harmful pathogens (26). Nutritional modulation has been proposed as an effective strategy to stimulate the mucosal innate immune system (27). Probiotics may induce endogenous functional molecules, including HDPs, through microbe–host cell signaling, thus improving the intestinal microecology and modulating the immune response (28). There are two major HDP families in pigs, defensins and cathelicidins, and most members of both families are secreted by gastrointestinal epithelial cells (10). The stimulatory potential of probiotic strains on HDP expression in different species, including pigs, has been demonstrated (12, 13). However, the underlying signaling mechanisms remained not completely understood.

We investigated the inductive effects of *L. plantarum* ZLP001 on porcine HDP expression using *in vivo* and *in vitro* models. In piglets, the effects were tissue-specific; the *L. plantarum* ZLP001-treated group had significantly higher mRNA levels of *pBD2, pBD3, pEP2C*, and *pBD114* in the jejunum and ileum than those in the control group, whereas no significant changes were observed in the duodenum after *L. plantarum* ZLP001 treatment. In line with our findings, a previous study showed that *Lactobacillus reuteri* I5007 significantly inducted *pBD2* expression in the jejunum but not in the ileum or colon (13). In addition, we found significant induction of *PG1-5* in the duodenum and no induction of *pBD129* in the ileum besides others segments. This indicated that different genes respond differentially to *L. plantarum* ZLP001 treatment in various intestinal tissue types, which might be due to gene- and cell type-specific differences in various porcine intestinal tissue types (10). Gene- and cell type-specific HDP induction by *L. plantarum* ZLP001 was verified by *in vitro* findings in IPEC-J2 and 3D4/31 cells. Although pEP2C showed the highest mRNA induction response among the six HDPs detected in both cell types, the other HDPs showed markedly different responses. Such specific regulation has also been demonstrated in other species and with other stimulators (10, 29, 30). In addition, it is well-known that the effect of probiotics is strain-specific in most cases. Different *Lactobacillus* strains also showed varying capacities in inducing a human HDP (hBD-2) mRNA expression (12). Comparative studies of multiple *Lactobacillus* strains on porcine HDP induction are needed in the future.

The innate immune system initiates a response to microorganisms *via* pattern recognition receptors (PRRs), which have been extensively studied to explain the regulatory effects of immunobiotic bacteria in both immune and intestinal epithelial cells (31). TLR2 is an important PRR that plays an essential role in immune regulation by probiotics. It has a broad recognition profile that includes products from gram-positive bacteria, including lipoteichoic acid, peptidoglycan, lipoproteins, and so on (32). TLR2 is essential to human HDP DEFB131 production after lipoteichoic acid treatment, as evidenced by the result that TLR2-silenced RWPE-1 cells exhibited diminished DEFB131 production (33). We found that TLR2-silenced IPEC-J2 cells show decreased pBD2 mRNA expression and secretion upon *L. plantarum* ZLP001 treatment, which indicates that *L. plantarum* ZLP001 exerts an HDP production-inductive effect through TLR2 recognition of its products. Similarly, Schlee et al. (12) suggested that *Lactobacillus* strains induce human hBD-2 likely *via* a bacterial cell wall component, as *Lactobacillus* pellets showed similar *hBD-2*-inductive capacity as culture media. In contrast, in a study using the probiotic strain *Escherichia coli* Nissle 1917, culture supernatant exhibited a 13-fold stronger *hBD-2*-inductive effect than pelleted bacteria (34). We have confirmed that live bacteria of *L. plantarum* ZLP001 exhibit highest induction effect on HDP expression compared with other different processing bacteria, including bacterial free culture supernatant, in our preliminary experiment. However, which bacterial cell component or products of *L. plantarum* ZLP001 are recognized by TLR2 and provoked subsequent responses responsible for the stimulation of HDP still need to be clarified in further studies. In addition, whether TLR2 exerts its actions by forming heterodimers with other TLRs (e.g., TLR1 or TLR6) (35, 36) and cooperating with TLR2 coreceptors (37) remains to be investigated.

TLR2 activation triggers signaling pathways involving MAPKs and transcription factors. MAPKs, including the three major MAPKs, ERK, p38, and JNK, regulate various processes associated with innate immunity and the proinflammatory response, as well as with cell metabolism and transcription (38, 39). The family of MAPK pathways is linked to HDP expression by various stimuli in different species (34, 40, 41). For example, human cathelicidin LL-37 expression induced by short-chain fatty acids is mediated by the MEK/ERK pathway, and branched-chain amino acids can activate ERK1/2 and the downstream transcription factor 90RSK to induce porcine β-defensin expression (41, 42). On the other hand, epigallocatechin-3-gallate induced *pBD2* expression *via* p38, not ERK or JNK signaling (43). In the present study, ERK1/2 and JNK were clearly activated during *L. plantarum* ZLP001-induced HDP expression but not p38, and the involvement of these two MAPKs in HDP expression induction was corroborated by pharmacological inhibition experiments. In line with our findings, a previous study on human *hBD-2* gene expression upon Caco-2 cells after treatment with the probiotics *Lactobacillus fermentum, Lactobacillus acidophilus*, and *Pediococcus pentosaceus* revealed that ERK1/2, JNK, and p38 were apparently activated by these strains, although p38 seemed less important to the regulation of hBD-2 (12). However, studies of the probiotic strain *Escherichia coli* 1917 demonstrated that its inductive effect on *hBD-2* was mediated by the JNK pathway, not the ERK1/2 or p38 signaling (34). Our and previous findings clearly indicate that probiotics modulate immune functions against pathogens in a species- and strain-specific manner (44). Furthermore, the present study revealed that ERK1/2 and JNK inhibitors had differential effects on different HDP genes, indicating that both ERK1/2 and JNK separately contribute to each porcine HDP regulation. This suggests that the mechanisms underlying the regulation of different HDP genes are different perhaps principally owing to different promoter structures and transcription factor-binding sites. Certainly, the possibility that other signaling pathways participate in HDP induction cannot be excluded based on our present results. Specific recognition mechanisms and other pathways should be addressed in future investigations.

*L. plantarum* ZLP001-induced upregulation of HDPs in pigs may involve a transcriptional mechanism. However, the molecular mechanisms involved in the transcriptional modulation of HDPs are not well-understood. The AP-1 transcription factor complex is the main transcription factor downstream of MAPK pathways and can bind with specific DNA sequences (45, 46). In the present study, the AP-1 subunits c-fos and c-jun were activated in IPEC-J2 cells incubated with *L. plantarum* ZLP001. They may bind to specific porcine HDP promoter DNA consensus sequences to initiate transcription. DNA-binding activity is an important capacity of transcription factors for transcriptional regulation. Our further studies corroborated that *L. plantarum* ZLP001 promotes c-fos and c-jun DNA-binding activities. These results confirmed the important role of AP-1 in the stimulation of porcine HDP expression in response to *L. plantarum* ZLP001. The study by Schlee et al. (12) also suggested that binding of AP-1 to the *hBD-2* promoter plays an essential role in *hBD-2* promoter activation initiated by *L. fermentum* and *P. pentosaceus*. Further investigations will be needed to explore the promoter region of each porcine HDP gene and find its specific binding site for transcription factors downstream of MAPK or other signaling pathways that play essential roles in the regulation of HDP expression.

## Conclusion

**Figure 7:**
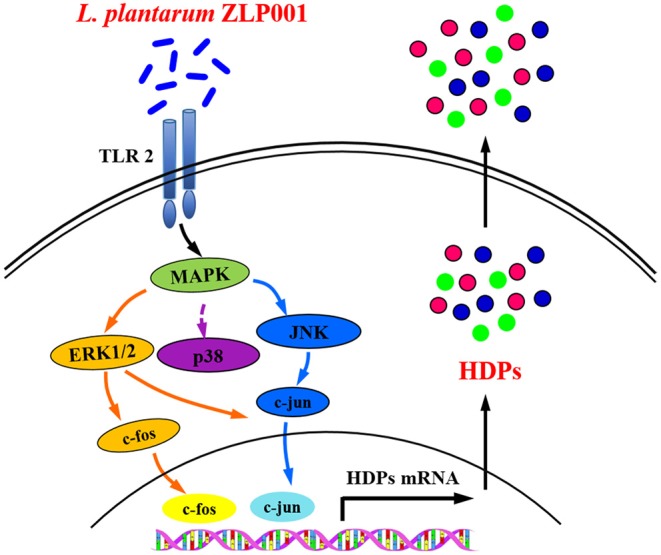
Suggested mechanism by which *L. plantarum* ZLP001 modulates porcine intestinal endogenous host defense peptide (HDP) synthesis. *L. plantarum* ZLP001 may induce porcine HDP expression and secretion through toll-like receptor (TLR)2 recognition as well as extracellular signal-regulated kinase (ERK)1/2/c-jun N-terminal kinase (JNK) and c-jun/c-fos pathway activation.

This study revealed that *L. plantarum* ZLP001 supplementation induces porcine HDP expression and production *in vivo* and *in vitro*. Furthermore, our study provided insights into the mechanism underlying *L. plantarum* ZLP001-induced porcine HDP expression (Figure 7). Upon *L. plantarum* ZLP001 stimulation, TLR2 recruits signaling molecules to intracellular signaling pathways within IPEC-J2 cells, leading to ERK1/2/JNK and c-fos/c-jun activation. We believe that the induction of defensins by probiotics such as *L. plantarum* ZLP001 might be an interesting new dietary strategy for strengthening innate defense function in weaning piglets. Further studies should focus on identifying bacterial cell wall components that play major roles in this inductive process and the specific signaling pathways regulating specific HDPs.

## Data Availability Statement

All datasets generated for this study are included in the article/Supplementary Material.

## Ethics Statement

The animal study was reviewed and approved by Animal Care and Use Committee of Institute of Animal Husbandry and Veterinary Medicine of the Beijing Academy of Agriculture and Forestry Sciences. Written informed consent was obtained from the owners for the participation of their animals in this study.

## Author Contributions

JW and HJ conceived and designed the study. JW, WZ, SW, HL, and DZ performed the experiments. JW and WZ analyzed the data and drafted the manuscript. YW contributed reagents and materials. All authors read and approved the final manuscript.

### Conflict of Interest

The authors declare that the research was conducted in the absence of any commercial or financial relationships that could be construed as a potential conflict of interest.
