# Supplementary material for: Swine-Derived Probiotic Lactobacillus plantarum Modulates Porcine Intestinal Endogenous Host Defense Peptide Synthesis Through TLR2/MAPK/AP-1 Signaling Pathway
Source: Front Immunol. 2019 Nov 19;10:2691. doi: 10.3389/fimmu.2019.02691 (PMC6877743; doi:10.3389/fimmu.2019.02691)
Supplement: Supplementary file 1 [file Table_1.docx]

**Supplementary Table S1** Sequences of the primers used in this study.

| Gene | Forward primer | Reverse primer | Product size (bp) |
| --- | --- | --- | --- |
| *GAPDH* | GCTACACTGAGGACCAGGTTG | CCTGTTGCTGTAGCCAAATTC | 146 |
| *TLR2* | TCACTTGTCTAACTTATCATCCTC | TCAGCGAAGGTGTCATTATTGC | 162 |
| *pBD2* | TGTCTGCCTCCTCTCTTCC | AACAGGTCCCTTCAATCCTG | 149 |
| *pBD3* | CCTTCTCTTTGCCTTGCTCTT | GCCACTCACAGAACAGCTACC | 163 |
| *PG1-5* | GTAGGTTCTGCGTCTGTGTCG | CAAATCCTTCACCGTCTACCA | 166 |
| *pEP2C* | ACTGCTTGTTCTCCAGAGCC | TGGCACAGATGACAAAGCCT | 92 |
| *pBD114* | TGTACCTTGGTGGATCCTGAACGA | CGCCCTCTGAATGCAGCATATCTT | 240 |
| *pBD129* | CAAAGACCACTGTGCCGTGAATGA | TTGATGCTGGCGAAAGGGTTGGTA | 131 |

**Supplementary Table S2** Antibodies used in this study.

| Antibody | Supplier | Dilution |
| --- | --- | --- |
| Rabbit polyclonal anti-p38 MAPK (Cat number: #9212) | Cell Signaling Technology, Danvers, MA, USA | 1:1,000 |
| Rabbit monoclonal anti-p-p38 MAPK (Cat number: #4511) | Cell Signaling Technology | 1:1,000 |
| Rabbit monoclonal anti-ERK1/2 MAPK (Cat number: #4695) | Cell Signaling Technology | 1:1,000 |
| Rabbit monoclonal anti-p-ERK1/2 MAPK (Cat number: #4370) | Cell Signaling Technology | 1:1,000 |
| Rabbit monoclonal anti-JNK (Cat number: #9258) | Cell Signaling Technology | 1:1,000 |
| Rabbit monoclonal anti-p-JNK (Cat number: #4668) | Cell Signaling Technology | 1:1,000 |
| Rabbit monoclonal anti-c-Jun (Cat number: #9165) | Cell Signaling Technology | 1:1,000 |
| Rabbit monoclonal anti-p-c-Jun (Cat number: #2361) | Cell Signaling Technology | 1:1000 |
| Rabbit monoclonal anti-TLR2 (Cat number: #12276) | Cell Signaling Technology | 1:1,000 |
| Rabbit monoclonal anti-c-fos (Cat number: #YM3241) | Immunoway, Plano, TX, USA | 1:1,000 |
| Rabbit monoclonal anti-p-c-fos (Cat number: #YP0042) | Immunoway | 1:1,000 |
| Mouse monoclonal anti-β-actin (Cat number: #YM3028) | Immunoway | 1:10,000 |
